# Neoadjuvant chemotherapy improves overall survival in resectable colorectal liver metastases patients with high clinical risk scores—— A retrospective, propensity score matching analysis

**DOI:** 10.3389/fonc.2022.973418

**Published:** 2022-09-05

**Authors:** Feng-Lin Chen, Yan-Yan Wang, Wei Liu, Bao-Cai Xing

**Affiliations:** Key Laboratory of Carcinogenesis and Translational Research, Ministry of Education, Peking University School of Oncology, Hepatopancreatobiliary Surgery Department I, Beijing Cancer Hospital and Institute, Beijing, China

**Keywords:** colorectal cancer, liver metastasis, hepatectomy, neoadjuvant chemotherapy, survival

## Abstract

**Background:**

The use of neoadjuvant chemotherapy (NAC) in resectable colorectal liver metastases (CRLM) patients is controversial. High-risk patients are more likely to benefit from NAC despite its hepatotoxic effects. Since patients with a high tumor burden receive NAC more frequently, previous retrospective studies have imbalanced baseline characteristics. The results of randomized controlled trials are still pending. This study aimed to assess the efficacy of NAC in resectable CRLM patients with high clinical risk scores (CRS) proposed by Fong et al. after balancing baseline characteristics by propensity score matching (PSM).

**Methods:**

Resectable CRLM patients with high CRS (3-5) undergoing hepatectomy between January 2003 and May 2021 were retrospectively studied. Patients were divided into the NAC and the upfront surgery group. Survival outcomes and surgical outcomes were compared after PSM.

**Results:**

The current study included 322 patients with a median follow-up of 40 months. After one-to-two PSM, patients were matched into the upfront surgery group (n = 56) and the NAC group (n = 112). Baseline characteristics were balanced after matching. There was no difference in long-term progression-free survival (PFS), while overall survival (OS) from the initial diagnosis was improved in the NAC group (*P* = 0.048). Postoperative hospital stays were shorter in the NAC group (*P* = 0.020). Surgical outcomes were similar, including major hepatectomy rate, intraoperative ablation rate, blood loss, operative time, perioperative blood transfusion, positive surgical margin, and postoperative intensive care unit stay. In multivariable analysis, *RAS* mutation, maximum tumor diameter≥3cm, and no NAC were independent risk factors for OS. The 1-year PFS in the NAC group was improved, although it failed to reach a statistical difference (*P* = 0.064).

**Conclusions:**

NAC could improve OS in resectable CRLM patients with high CRS (3-5) and have a shorter postoperative hospital stay.

## Introduction

Colorectal cancer (CRC) is the second leading cause of cancer-related death worldwide (1). Up to 50% of the patients will develop colorectal liver metastases (CRLM) during the disease course (2). Hepatic resection is the main radical treatment to achieve long-term survival (3). With the advances in systemic therapy, previously unresectable CRLM patients have a chance to undergo curative-intent surgery, and the outcomes have significantly improved (4–6).

However, the clinical benefit of neoadjuvant chemotherapy (NAC) in resectable CRLM patients is still under debate. The European Organization for Research and Treatment of Cancer (EORTC) trial 40983 was the only randomized controlled trial (RCT) that has been conducted regarding this. While they reported a prolonged progression-free survival (PFS) in patients treated with perioperative chemotherapy (7), the overall survival (OS) was not improved after a long-term follow-up (8). Notably, this trial only included low-risk patients. Patients with five or more liver metastases were excluded, and more than half of the patients only had a single tumor. Whereas in clinical practice, patients receiving NAC typically presented with a high-risk profile. Several retrospective analyses of high-risk patients have suggested that NAC could improve OS for resectable CRLM patients treated by liver resection (9–13). Even though these studies focused only on high-risk patients, there were still imbalances in baseline characteristics between patients treated by NAC and upfront surgery, which could reduce the reliability of their results.

Propensity score matching (PSM) analysis is one possible way to reduce the treatment assignment bias and emulate randomization in observational studies (14). Fong’s clinical risk score (CRS) is the most widely used and validated risk scoring system (15). Patients were stratified into the low-risk group (CRS 0-2) and the high-risk group (CRS 3-5) based on five prognostic factors. In the current study, we analyzed the survival outcomes of high-risk patients by Fong’s CRS. This study aimed to evaluate the efficacy of NAC in the high-risk subset after adjusting baseline characteristics by PSM analysis.

## Methods

### Study population

The current study retrospectively included CRLM patients who underwent liver resection between January 2003 and May 2021 at the Hepatopancreatobilary Surgery Department I of Peking University Cancer Hospital. All data were collected prospectively and analyzed retrospectively. All patients included in the study signed a written consent form. The study was examined and certified by the Ethics Committee of Beijing Cancer Hospital and performed according to the Declaration of Helsinki.

The inclusion criteria were as follows: 1) pathologically confirmed CRLM; 2) considered a resectable disease by a multidisciplinary team (MDT) at diagnosis; 3) CRS >2 at diagnosis; 4) no evidence of extrahepatic disease; 5) complete destruction of the liver metastases and primary site, either by surgical removal alone, or combined with intraoperative radiofrequency ablation (RFA) or postoperative stereotactic body radiotherapy (SBRT). According to the criteria, this retrospective observational study included 322 patients.

### Disease management

Abdominal and pelvic enhanced computed tomography (CT) scan, chest CT and abdominal enhanced magnetic resonance imaging (MRI) were performed to evaluate resectability and detect extrahepatic disease. Resectability was defined as the complete destruction of the tumor, whether by surgical removal alone or surgery combined with intraoperative RFA or postoperative SBRT, with a sufficient remnant liver function. The indication for NAC was decided by the MDT discussion, and the treatment policy changed over time with the understanding of CRLM in our center. Patients were re-evaluated after chemotherapy. If the disease was not controlled, second-line chemotherapy was given. Parenchymal-sparing liver resections were performed in principal. More than 40% of the remnant volume was preserved in patients with chemotherapy injuries (16). Patients received perioperative chemotherapy for 6 months if considered necessary by the treatment team, unless contraindicated due to poor tolerance, comorbidities, or patients’ own will. All patients were followed up every 3 months for 2 years after liver resection, then every 6 months. Follow-up included abdominal and pelvic enhanced CT, chest CT and measurement of tumor biomarker levels.

### Clinicopathologic characteristics and long-term outcomes

Primary tumor T stage and primary tumor lymph node status were collected from the pathological reports of the resected specimen of the primary tumor. Other clinicopathologic characteristics were collected at the initial clinical diagnosis of CRLM. OS was defined as the interval from the date of clinical diagnosis of liver metastases to the date of death. PFS was defined as the interval from the date of clinical diagnosis of liver metastases to the date of recurrence.

### Propensity score matching

Propensity score matching was performed to reduce selection bias by establishing comparable patient cohorts concerning factors associated with receipt of neoadjuvant chemotherapy. Propensity scores were estimated using a logistic regression model based on patients’ gender, age, primary tumor site, primary tumor T stage, primary tumor lymph node status, *RAS* mutation, disease-free interval, number of liver metastases, distribution of liver metastases, maximum tumor diameter and carcinoembryonic antigen (CEA) level. One-to-two matching was performed using a 0.05 caliper width. Unmatched patients and patients with scores outside the caliper were excluded.

### Statistical analysis

Categorical variables were summarized as frequency and percentage and were compared using the chi-square test. Non-normal distributed continuous variables were presented as median with interquartile ranges (IQR) and were compared using the Mann-Whitney *U*-test. Survival curves were calculated by the Kaplan–Meier method and compared by the log-rank test. Univariate and multivariate analyses of clinicopathological factors were performed by Cox’s proportional hazard model to identify independent prognostic factors. A two‐tailed *P* value less than 0.05 was considered to be statistically significant. The analysis was done using R, version 4.1.0 (www.r-project.org).

## Results

### Patient demographics before and after PSM

Among the 322 patients included in the study, 60/322 (18.6%) patients received upfront surgery, and 262/322 (81.4%) received NAC. The median follow-up period was 40 months. Propensity scoring was used to match the patients into an upfront surgery group of 56 patients and a NAC group of 112 patients. In the matched cohort, baseline characteristics, including gender, age, primary tumor site, primary tumor T stage, primary tumor lymph node status, *RAS* mutation, disease-free interval, number of liver metastases, distribution of liver metastases, maximum tumor diameter, and CEA level were all balanced. The baseline demographics of the unmatched and matched cohorts are summarized in **Table 1**.

**Table 1:** Baseline characteristics for patients with upfront surgery or neoadjuvant chemotherapy before and after Propensity Score Matching.

	Overall cohort			Matched cohort			
Characteristic	Upfront surgery	Neoadjuvant chemotherapy	*p*-Value	Upfront surgery	Neoadjuvant chemotherapy	*p*-Value	
	(n=60)	(n=262)		(n=56)	(n=112)		
Gender						
Female	23 (38.33)	85 (32.44)	0.471	22 (39.29)	38 (33.93)	0.608^a^	
Male	37 (61.67)	177 (67.56)		34 (60.71)	74 (66.07)		
Age						
<60	34 (56.67)	146 (55.73)	1.000	32 (57.14)	61 (54.46)	0.869^a^	
≥60	26 (43.33)	116 (44.27)		24 (42.86)	51 (45.54)		
Primary site						
Left	44 (73.33)	222 (84.73)	0.056	43 (76.79)	90 (80.36)	0.737^a^	
Right	16 (26.67)	40 (15.27)		13 (23.21)	22 (19.64)		
Primary tumor T stage						
T_1-2_	4 (6.67)	16 (6.11)	1.000	3 (5.36)	7 (6.25)	1.000^a^	
T_3-4_	56 (93.33)	246 (93.89)		53 (94.64)	105 (93.75)		
Primary tumor lymph node status						
Negative	2 (3.33)	43 (16.41)	0.015	2 (3.57)	3 (2.68)	1.000^a^	
Positive	58 (96.67)	219 (83.59)		54 (96.43)	109 (97.32)		
*RAS* mutation						
Wild	42 (70.00)	152 (58.02)	0.118	38 (67.86)	86 (76.79)	0.292^a^	
Mutation	18 (30.00)	110 (41.98)		18 (31.54)	26 (23.21)		
Disease-free interval						
≥12m	1 (1.67)	5 (1.91)	1.000	1 (1.79)	1 (0.89)	1.000^a^	
<12m	59 (98.33)	257 (98.09)		55 (98.21)	111 (99.11)		
Number of liver metastases						
Single	11 (18.33)	29 (11.07)	0.186	8 (14.29)	12 (10.71)	0.674^a^	
Multiple	49 (81.67)	233 (88.93)		48 (85.71)	100 (89.29)		
Distribution of liver metastases						
Unilobar	22 (36.67)	96 (36.64)	1.000	20 (35.71)	42 (37.50)	0.955^a^	
Bilobar	38 (63.33)	166 (63.36)		36 (64.29)	70 (62.50)		
Maximum tumor diameter						
<3cm	31 (51.67)	125 (47.71)	0.682	31 (55.36)	64 (57.14)	0.956^a^	
≥3cm	29 (48.33)	137 (52.29)		25 (44.64)	48 (42.86)		
CEA level (ng/ml)						
<30	40 (66.67)	145 (55.34)	0.146	37 (66.07)	77 (68.75)	0.861^a^	
≥30	20 (33.33)	117 (44.66)		19 (33.93)	35 (31.25)		

RAS, rat sarcoma viral oncogene homolog; CEA, carcinoembryonic antigen.

a These variables were compared using the chi-square test.

### Chemotherapy details, surgical outcomes, and recurrence details after PSM

The chemotherapy details, surgical outcomes, and recurrence details of matched patients who received NAC and upfront surgery were reported in **Table 2**. In the NAC group, 65/112 (58.04%) patients received an Oxaliplatin-based regimen, and 38/112 (33.93%) patients received an Irinotecan-based regimen. Adjuvant chemotherapy was performed in 83/112 (74.11%) patients in the NAC group and 36/56 (64.29%) patients in the upfront surgery group. Postoperative hospital stay was significantly shorter in patients treated by NAC. No other differences were observed in surgical outcomes. After matching, the site of recurrence and treatment of recurrence also showed no difference between groups.

**Table 2:** Chemotherapy details, surgical outcomes, postoperative complications, and recurrence details for patients with upfront surgery or neoadjuvant chemotherapy after Propensity Score Matching analysis.

Variables	Upfront surgery	Neoadjuvant chemotherapy	*p*-Value
	(n=56)	(n=112)	
**Chemotherapy details**				
Chemotherapy regimen				
Oxaliplatin-based	NA	65 (58.04)		NA
Irinotecan-based	NA	38 (33.93)		NA
Oxaliplatin+Irinotecan	NA	5 (4.46)		NA
Other	NA	4 (3.57)		NA
Use of biological agents				
Bevacizumab	NA	33 (29.20)		NA
Cetuximab	NA	37 (32.74)		NA
Chemotherapy cycles				
< 6 cycles	NA	87 (77.68)		NA
≥ 6 cycles	NA	23 (20.54)		NA
Unknown	NA	2 (1.78)		NA
No. of chemotherapy lines				
First line	NA	99 (88.39)		NA
Other	NA	13 (11.61)		NA
Adjuvant chemotherapy	36 (64.29)	83 (74.11)		0.254^a^
**Surgical outcomes**				
Major hepatectomy	10 (17.86)	24 (21.43)		0.734^a^
Intraoperative ablation	7 (12.50)	25 (22.32)		0.187^a^
Blood loss (ml) (median[IQR])	200[100, 200]	200[100, 200]		0.736^b^
Operative time (min) (median[IQR])	183[154.50, 229]	208[159.25, 242]		0.206^b^
Perioperative RBC transfusion	7 (12.50)	6 (5.36)		0.184^a^
Surgical margin				0.230^a^
R0	52 (92.86)	94 (83.93)		
R1	4 (7.14)	16 (14.29)		
R2	0 (0.00)	2 (1.79)		
Postoperative hospital stay (days) (median[IQR])	10[7, 14]	7[7, 11]		0.020^b^
Postoperative ICU stay	1 (1.79)	2 (1.79)		1.000^a^
**Recurrence details**				
Site of recurrence				
Liver only	20 (35.71)	33 (29.46)		0.519^a^
Others	5 (8.93)	11 (9.82)		1.000^a^
Multiple	15 (26.79)	29 (25.89)		1.000^a^
Treatment of recurrence				
Resection	10 (17.86)	17 (15.18)		0.824^a^
Other	30 (53.57)	56 (50.00)		0.785^a^

RBC, red blood cell; IQR, interquartile range; ICU, intensive care unit.

a These variables were compared using the chi-square test.

b These variables were compared using the Mann-Whitney U-test.

### Survival outcomes of patients before and after PSM

**Figure 1:**
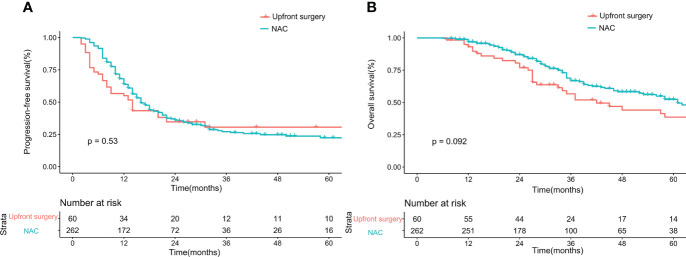
Kaplan-Meier curves for progression-free survival **(A)** and overall survival **(B)** between upfront surgery and neoadjuvant chemotherapy groups before 1:2 Propensity Score Matching Analysis. NAC, neoadjuvant chemotherapy.

**Figure 2:**
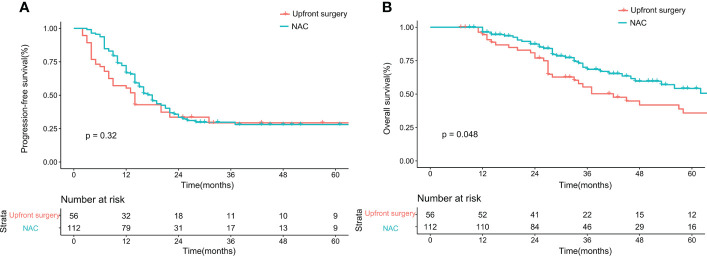
Kaplan-Meier curves for progression-free survival **(A)** and overall survival **(B)** between upfront surgery and neoadjuvant chemotherapy groups after 1:2 Propensity Score Matching Analysis. NAC, neoadjuvant chemotherapy.

Before matching, the PFS showed no difference between the upfront surgery group and the NAC group (*P* = 0.53) (**Figure 1A**). For OS, although the survival curve of upfront surgery seemed worse than the NAC group, the difference was not statistically significant (*P* = 0.092) (**Figure 1B**). After matching, the PFS still showed no difference between groups (*P* = 0.32) (**Figure 2A**), but the OS of the NAC group was better than the upfront surgery group (*P* = 0.048) (**Figure 2B**). The 1-year, 3-year, and 5-year OS rates after PSM were 96.4%, 68.5%, and 50.6% in the NAC group, 94.4%, 50.2%, and 35.8% in the upfront surgery group, respectively.

### Univariate and multivariate analysis of OS after PSM

The results of univariate and multivariate analyses of variables associated with OS were summarized in **Table 3**. In univariate analysis, *RAS* mutation, maximum tumor diameter ≥ 3cm, and NAC were associated with worse OS. In multivariate analysis, these three factors were proved to be independent risk factors for OS.

**Table 3:** Results of univariate and multivariate Cox regression analysis for overall survival in patients with upfront surgery or neoadjuvant chemotherapy after Propensity Score Matching analysis.

Characteristic	Univariate analysis		Multivariate analysis	
	HR (95% CI)	*p*-Value	HR (95% CI)	*p*-Value
Gender				
Female	Ref		Not entered	
Male	0.726 (0.447-1.177)	0.194		
Age				
<60	Ref		Not entered	
≥60	0.770 (0.471-1.261)	0.299		
Primary site				
Left	Ref		Not entered	
Right	1.142 (0.650-2.007)	0.644		
Primary tumor T stage				
T_1-2_	Ref		Not entered	
T_3-4_	1.163 (0.365-3.710)	0.798		
Primary tumor lymph node status				
Negative	Ref		Not entered	
Positive	0.494 (0.155-1.577)	0.234		
*RAS* mutation				
Wild	Ref		Ref	
Mutation	2.068 (1.251-3.421)	0.005	1.894 (1.131-3.170)	0.0151
Disease free interval				
≥12m	Ref		Ref	
<12m	0.298 (0.072-1.229)	0.094	0.331 (0.08-1.379)	0.129
Number of liver metastases				
Single	Ref		Not entered	
Multiple	1.174 (0.536-2.571)	0.688		
Distribution of liver metastases				
Unilobar	Ref		Not entered	
Bilobar	0.985 (0.602-1.611)	0.952		
Maximum tumor diameter				
<3cm	Ref		Ref	
≥3cm	2.051 (1.263-3.330)	0.004	1.975 (1.172-3.329)	0.011
CEA level (ng/ml)				
<30	Ref		Ref	
≥30	1.471 (0.900-2.406)	0.124	0.989 (0.578-1.691)	0.967
Neoadjuvant chemotherapy				
No	Ref		Ref	
Yes	0.615 (0.379-0.998)	0.049	0.612 (0.377-0.992)	0.046

RAS, rat sarcoma viral oncogene homolog; CEA, carcinoembryonic antigen.

### One year PFS after PSM

**Figure 3:**
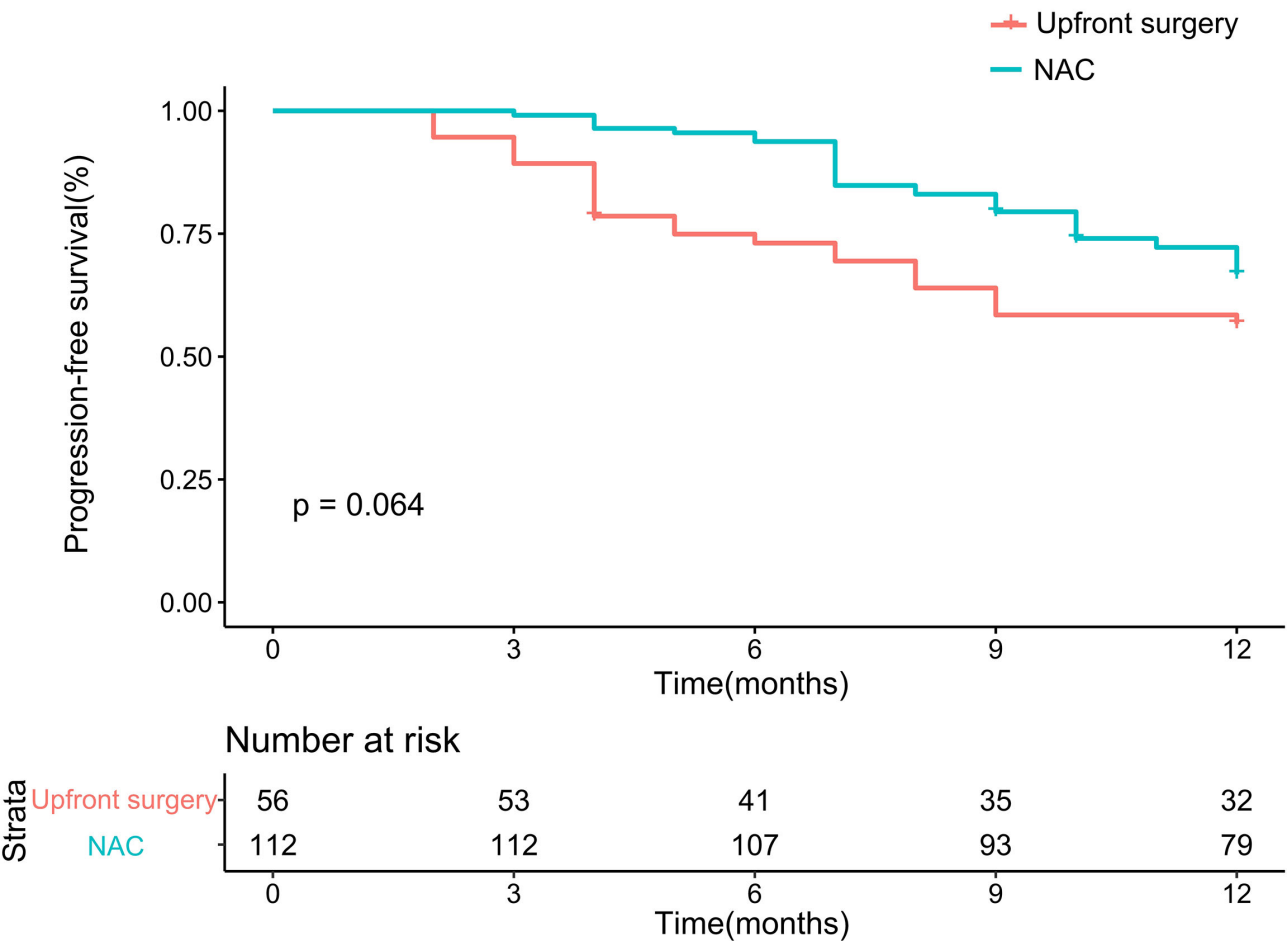
Kaplan-Meier curves for 1-year progression-free survival between upfront surgery and neoadjuvant chemotherapy groups after 1:2 Propensity Score Matching Analysis. NAC, neoadjuvant chemotherapy.

After matching, the 1-year PFS seemed better in the NAC group on the Kaplan-Meier curve. Therefore, we further analyzed 1-year PFS. The NAC group showed better survival than the upfront surgery group. The 1-year PFS rates were 57.1% in the upfront surgery group and 70.5% in the NAC group, respectively. The *p*-Value failed to reach a statistical difference (*P* = 0.064, **Figure 3**).

## Discussion

Surgical resection is the main radical treatment for resectable CRLM. However, the benefit of NAC is still under debate. The current retrospective study was the first one focusing on high-risk resectable CRLM patients and used PSM to adjust the imbalanced characteristics between patients who received upfront surgery and NAC. This study suggested that resectable CRLM patients with a high-risk profile could benefit from NAC. Compared to upfront surgery, NAC could provide a prolonged OS while having a shorter postoperative hospital stay.

For patients presenting with resectable CRLM upon diagnosis, the benefit of NAC remains controversial. The EORTC 40983 reported an increase in 3-year PFS but no significant improvement in OS (8). However, this study only included patients with a low-risk profile, approximately half of the study participants had a single or metachronous tumor. Previous study suggested that patients with a low CRS benefit less from adjuvant therapy than patients with a high CRS (17). Similarly, in EORTC 40983, the benefit of NAC might be underestimated. For high-risk patients, several retrospective studies have examined the effects of NAC and reported a prolonged OS in NAC group (9–12, 18). Since NAC was more frequently given to high-risk patients in clinical practice, there were imbalances in baseline characteristics between subgroups. For powerful RCTs, such as the CHARISMA trial, the results are still pending (19). To further investigate the efficacy and safety of NAC, this retrospective propensity score matching analysis was conducted. High-risk CRLM patients presented with a worse prognosis and were most likely to benefit from perioperative chemotherapy. However, only a limited number of studies were focused on these patients, and no high evidence level study was published. Therefore, we selected high-risk patients and further performed PSM to adjust the imbalances between study groups. As a high-volume center, with the treatment policy varied over time, we were able to create a study cohort with balanced baseline characteristics. By eliminating differences between groups using PSM, we provided new insights on the efficacy of NAC.

In the current study, NAC presented a beneficial effect of prolonged OS in the matched cohort. At the same time, the 1-year PFS was also improved in the NAC group. Due to the limited sample size after matching, the *p*-Value of 1-year PFS failed to reach a statistical significance (*P* = 0.064). Potential benefits of NAC include early treatment of micrometastatic disease, identifying patients who respond well to chemotherapy, and tumor downstaging (20). In our study, the improvement of 1-year PFS might be from the early treatment of micrometastatic disease, thus delaying tumor progression. However, the long-term progression rate was similar between subgroups, which indicated that NAC could not eliminate but only delay tumor progression. Ayez et al. (10) reported an improvement in disease-free survival (DFS) in the NAC group after a median follow-up of 47 months in patients with CRS > 2. In this study, none of the patients received standard adjuvant chemotherapy after liver resection. While in our study, 36/56 (64.29%) patients in the upfront surgery group and 83/112 (74.11%) patients in the NAC group received adjuvant chemotherapy. For high-risk patients, the additional use of adjuvant chemotherapy could reduce recurrence (17). Adjuvant chemotherapy might be able to reduce micrometastases remedially in high-risk patients who received upfront surgery. Current guidelines recommend 6 months of perioperative chemotherapy for most CRLM patients undergoing surgical resection. We demonstrated the benefit of NAC in a more clinical setting. Our study also revealed that patients treated with NAC presented with a prolonged OS. Several retrospective studies have reported similar results (9–12, 18, 21), but its mechanism is still uncertain. Ayez et al. (10) reported that in patients treated by NAC, the recurrent disease was mostly limited to the liver and had a higher chance of being treated by curative intent. Similarly, a large, retrospective study conducted by Buisman et al. (21) also reported a decreased pulmonary recurrences rate. On the other hand, Yonekawa et al. (13) reported a similar recurrence rate, sites, and re-resection rate between patients treated with and without NAC. In the current study, the site of recurrence and treatment of recurrence failed to show statistical differences after matching. The negative result on recurrence patterns might be due to the balanced baseline risk factors by PSM, the different definitions of high-risk patients, or the limited sample size. In our study, the possible reason for prolonged OS might be due to the tumor control and elimination of micrometastasis by NAC, therefore delaying progression and converting to the beneficial effect on OS. Previous studies suggested that early progression is associated with worse survival outcomes, whether during neoadjuvant chemotherapy, chemotherapy to liver resection interval or after liver resection (22–27). As our study only included patients who completed liver resection, patients who were inoperable due to tumor progression in neoadjuvant therapy were not included. The improvement of PFS might be overestimated. However, only a limited number of patients were inoperable due to tumor progression (4.68% in EORTC 40983) (7), suggesting that the results were only mildly affect by the inclution creteria. Nevertheless, the exact mechanism of extended OS in high-risk patients brought by NAC still needs further investigation.

While demonstrating an improvement in OS by NAC, our study also revealed a shorter postoperative hospital stay in the NAC group. Other surgical outcomes did not have a significant difference. NAC could decrease tumor burden, therefore allowing a less extensive surgical procedure and speeding up postoperative recovery (20). However, a retrospective PSM study conducted by Wiseman et al. (28) reported that patients treated by surgery alone have a shorter postoperative hospital stay. The difference in risk profiles of study groups may result in such discrepancies. In high-risk patients, the benefit of narrowing the scope of surgery might be more significant, allowing a shorter postoperative hospital stay. Chemotherapy agents were reported to have hepatotoxic effects, which have a negative effect on patient outcome (29). While in high-risk patients, after adjusting baseline characteristics by PSM, no differences in major hepatectomy rate, intraoperative ablation, blood loss, operative time, perioperative RBC transfusion, surgical margin, and postoperative ICU stay were observed. Another multi-institutional study focusing on high-risk patients from Japan reported similar results (12). NAC could be a safe option for high-risk patients.

In multivariate analysis, *RAS* mutation, maximum tumor diameter, and no neoadjuvant chemotherapy were independent risk factors for OS. These results were aligned with previous studies. Ren et al. (30) reported that in the neoadjuvant setting, the size of the largest CRLM was an independent prognostic factor for survival outcomes. *RAS* mutation was reported to be a negative prognostic factor for recurrence and OS in patients receiving NAC (31). Currently, there is no consensus on the criteria for patients who would benefit from NAC. Future studies on patient selection for NAC could focus on these aspects.

For CRLM patients, liver resection is the main potentially curative treatment (32). Conversion chemotherapy was given to initially unresectable CRLM patients for tumor downstaging and to facilitate subsequent curative-intent surgery, while NAC was given to initially resectable patients for early treatment of micrometastatic disease, identifying patients who respond well to chemotherapy, and tumor downstaging (20). Notebly, the definition of resectability in CRLM patients varied, even between experienced liver surgeons (33). Preoperative chemotherapy was also recommended for sub-optimal resectable patients (34). Disagreements on use of NAC mainly exists in clearly resectable CRLM patients. Preoperative chemotherapy were associated with increased postoperative complication rates (35, 36), and the landmark EORTC 40983 trial did not report a significant improvement in OS from NAC (8). However, even in clearly resectable patients, there is a subgroup of high-risk patients exerts poor prognosis. These patients are most likely to benefit from NAC, but the study focused on them was rare. The EORTC 40983 trial mainly focused on low-risk patients, only several retrospective studies reported benefitial effects of NAC in high-risk resectable CRLM patients (9–12, 18, 21). Despite the lack of high-level evidence of efficacy of NAC in high-risk patients, current guidelines suggest preoperative treatment in resectable patients with unfavorable prognostic factors (34). Organizing clinical trials involving both surgery and chemotherapy is complex, and few RCTs have been completed (8, 19, 37). Only one of these was focused on high-risk patients, but the results are still pending (19). Our study used PSM to emulate the randomization process and further suggested that NAC is an efficient and safe treatment option in high-risk patients. Future studies could further focus on the criteria of selecting individual patients who could benefit from NAC most. Adjuvant chemotherapy has also been reported to have a beneficial effect (38, 39). Further studies on the optimal treatment sequence, duration, and regimen for high-risk patients are needed, which requires careful design since both preoperative and postoperative chemotherapy may affect patient outcomes. This study has the following limitations. Although we used PSM to reduce the imbalance between study groups, the retrospective nature of this study could raise potential selection bias. Since this is a retrospective analysis of surgical cases, patients who did not undergo surgery due to progressive disease or severe adverse events were omitted. Cautions should be paid in the actual clinical treatment decisions. All patients enrolled in this study were from a single center, which may induce bias. Our study ranged from 2003 to 2021. Although it allowed us to compare the long-term outcomes. The chemotherapy regimen changed during the long data collection period. The proportion of targeted therapies used in NAC increased in recent years. This change may reduce the reliability of our resuls. The small sample size of this study may decrease statistical power. Despite this, the current study is by far the largest PSM study regarding NAC in high-risk patients. With the use of PSM, we believe this retrospective study could provide insight into the benefit of NAC.

## Conclusion

This retrospective, propensity score matching analysis revealed that NAC is associated with improved OS and shorter postoperative hospital stay in patients with CRS > 2. And NAC did not increase postoperative complications.

## Data availability statement

The raw data supporting the conclusions of this article will be made available by the authors, without undue reservation.

## Ethics statement

The studies involving human participants were reviewed and approved by Ethics Committee of Beijing Cancer Hospital. The patients/participants provided their written informed consent to participate in this study.

## Author contributions

All authors contributed to the study conception and design. Material preparation, data collection, and analysis were performed by Chen F-L and Wang Y-Y. The first draft of the manuscript was written by Chen F-L and edited by Wang Y-Y. All authors commented on previous versions of the manuscript. All authors read and approved the final manuscript.

## Funding

This study was supported by Grants from the Beijing Capital’s Funds for Health Improvement and Research (CFH, No.2022-1-2151), Grants from the Beijing Hospitals Authority Clinical Medicine Development of Special Funding Support (code: ZYLX202116), Grants from the Beijing Hospitals Authority Youth Program (code: QMS20201105) and Scientific Research Fundation of Peking University Cancer Hospital (code: 2021-16).

## Acknowledgments

We acknowledge Kun Wang, Quan Bao, Hong-Wei Wang, Ke-Min Jin, Juan Li, who contributed to the study by making substantial contributions to the acquisition of the data.

## Conflict of interest

The authors declare that the research was conducted in the absence of any commercial or financial relationships that could be construed as a potential conflict of interest.

## Publisher’s note

All claims expressed in this article are solely those of the authors and do not necessarily represent those of their affiliated organizations, or those of the publisher, the editors and the reviewers. Any product that may be evaluated in this article, or claim that may be made by its manufacturer, is not guaranteed or endorsed by the publisher.
